# An Overview of Key Factors Affecting Genomic Selection for Wheat Quality Traits

**DOI:** 10.3390/plants10040745

**Published:** 2021-04-11

**Authors:** Ivana Plavšin, Jerko Gunjača, Zlatko Šatović, Hrvoje Šarčević, Marko Ivić, Krešimir Dvojković, Dario Novoselović

**Affiliations:** 1 Department for Cereal Breeding and Genetics, Agricultural Institute Osijek, Južno predgrađe 17, 31000 Osijek, Croatia; ivana.plavsin@poljinos.hr (I.P.); marko.ivic@poljinos.hr (M.I.); dario.novoselovic@poljinos.hr (D.N.); 2 Centre of Excellence for Biodiversity and Molecular Plant Breeding (CoE CroP-BioDiv), Svetošimunska cesta 25, 10000 Zagreb, Croatia; jgunjaca@agr.hr (J.G.); zsatovic@agr.hr (Z.Š.); hsarcevic@agr.hr (H.Š.); 3 Department of Plant Breeding, Genetics and Biometrics, Faculty of Agriculture, University of Zagreb, Svetošimunska cesta 25, 10000 Zagreb, Croatia; 4 Department of Seed Science and Technology, Faculty of Agriculture, University of Zagreb, Svetošimunska cesta 25, 10000 Zagreb, Croatia

**Keywords:** wheat quality, genomic selection, GEBV, prediction accuracy, training population, validation population, heritability

## Abstract

Selection for wheat (*Triticum aestivum* L.) grain quality is often costly and time-consuming since it requires extensive phenotyping in the last phases of development of new lines and cultivars. The development of high-throughput genotyping in the last decade enabled reliable and rapid predictions of breeding values based only on marker information. Genomic selection (GS) is a method that enables the prediction of breeding values of individuals by simultaneously incorporating all available marker information into a model. The success of GS depends on the obtained prediction accuracy, which is influenced by various molecular, genetic, and phenotypic factors, as well as the factors of the selected statistical model. The objectives of this article are to review research on GS for wheat quality done so far and to highlight the key factors affecting prediction accuracy, in order to suggest the most applicable approach in GS for wheat quality traits.

## 1. Introduction

According to estimates of the International Maize and Wheat Improvement Center (CIMMYT), the need for wheat (*Triticum aestivum* L.) and its products could increase by at least 50% until 2050 [1] as a result of extensive human population growth and dietary changes. Given the accelerated growth of the world’s population and the increased need for food production, the greatest emphasis in wheat breeding is placed on increasing grain yield. However, an increase in yield usually entails a decrease in protein content or grain quality [2,3]. Therefore, in breeding programs strong emphasis should be placed on improving grain quality [4]. In the context of wheat quality, the most important traits are grain protein content (GPC) and gluten content (GC), as they directly affect the technological properties of flour and dough [5,6,7]. The majority of these traits are characterized mostly by low heritability due to strong environmental impact [8,9,10].

The extensive development of high-throughput genotyping in the last couple of decades has led to the increasing use of molecular markers in plant breeding, which enabled the development of prediction methods based only on marker information such as genomic selection (GS) [11,12]. The first GS studies in wheat were published more than a decade ago [13,14]. The results of these studies showed that models based on genomic markers outperform models based only on pedigree relationships and that GS could successfully enhance rates of genetic gain, which provided a strong foundation for further research on GS in wheat. Later studies also showed that, if the traits of interest are complex and influenced by many quantitative trait loci (QTLs) each controlling a small proportion of phenotypic variation, GS will be more relevant than marker-assisted selection (MAS) [15,16].

Currently, the majority of researchers of GS in wheat consider grain yield and disease resistance as key traits for successful wheat production [17,18,19,20,21]. Such a strong focus on grain yield is understandable from a point of view where grain yield is not improving fast enough to fill the gap between production and projected demands in the near future [22]. Considering wheat’s role as the main ingredient in many different products fundamental to the nutrition of humankind, equal emphasis should be given to the quality traits, especially those related to the end-use quality of wheat products. An overview of GS research studies for traits such as grain yield, *Fusarium* head blight, stripe and brown rust resistance, plant height, days to heading, and preharvest sprouting (PHS) tolerance is given by Rutkoski et al. [23]. Despite their importance in the context of nutrition, research on GS for wheat quality traits is still scarce. In this context, the objectives of the present study are to review research on GS for wheat quality conducted so far and to highlight the key factors affecting GS accuracy in order to suggest the most applicable approach in GS for wheat quality traits.

## 2. Genomic Selection and Prediction Models Used

**Figure 1:**
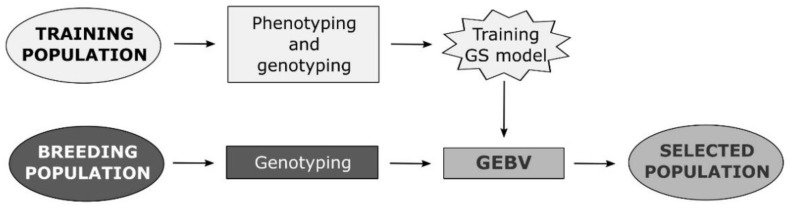
Flow diagram of a plant breeding program based on genomic selection.

Genomic selection is one of the newly developed methods that enables the prediction of breeding values of individuals by simultaneously incorporating all available marker information into a model [12]. Unlike other molecular breeding methods, GS does not require the identification of markers associated with QTLs of traits of interest. GS attempts to capture total additive genetic variance based on the sum of the effects of a large number of genetic markers, encompassing all QTLs that contribute to trait variability [24]. Therefore, the underlying genetic control in GS is not necessarily known. In GS, training population (TP) is genotyped using one of the methods of high-throughput genotyping and phenotyped for desired traits in a target set of environments. Obtained data are used to train a model that will be applied to the breeding population (BP) of unphenotyped individuals (selection candidates) to calculate their genomic-estimated breeding values (GEBVs) using only the marker scores [12] (Figure 1). The most important advantage of GS over phenotypic selection (PS) is the increase of genetic gain due to the shortening of the selection cycle in breeding process by reducing the need for phenotyping [25,26].

**Figure 2:**
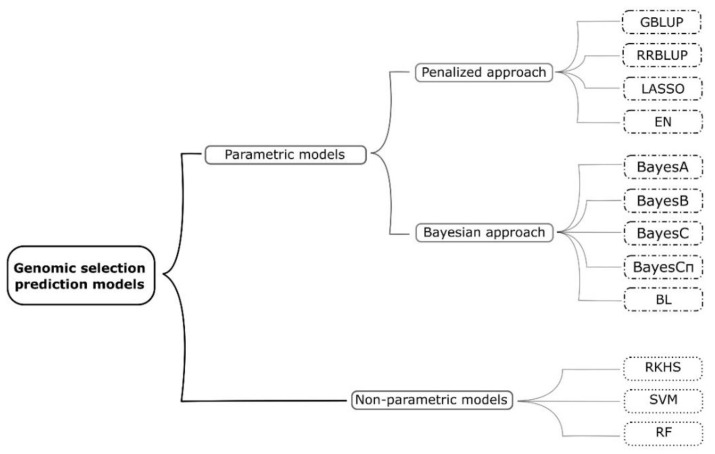
Classification of the most frequently used genomic selection prediction models.

High-throughput genotyping generates a large amount of marker data, which are then used in GS. When the number of predictor variables (markers) is much greater than the number of observations (phenotypic values), the result is an infinite number of marker effect estimates. In order to reduce the problem of highly dimensioned data, different parametric and nonparametric models have been developed and used in GS (Figure 2).

The prediction models differ mainly in the assumptions of the distribution of the marker effects, i.e., the assumptions of how the marker effects contribute to the overall variance [12]. Detailed features of GS models and their (dis)advantages are already given elsewhere [27,28,29], so they will be just briefly discussed here. Genomic best linear unbiased predictor (GBLUP) uses a genomic-estimated relationship matrix, assigns equal variance to all markers, and assumes that they are equally contributing to the trait of interest [29]. Ridge-regression best linear unbiased predictor (RRBLUP) assumes that all markers have common variance but allows that markers have unequal effects. RRBLUP shrinks all marker effects equally towards zero, regardless of the size of their effect, which can possibly lead to overshrinking of large-effect loci [12,15,30]. On the other hand, Bayesian alphabet models (BayesA, BayesB, BayesC, BayesCπ) assign different types of prior distribution to marker effects, thus having a more realistic assumption of marker effects [12,13,21]. LASSO (Least absolute shrinkage and selector operator) and Bayesian LASSO (BL) models use both variable selection and shrinkage methods, with the difference that BL additionally applies prior exponential distribution on marker variances [28]. Like RRBLUP, Bayesian ridge regression (BRR) shrinks marker effects equally towards zero but additionally uses prior Gaussian distribution for marker effects [31]. Elastic net (EN) uses two penalty methods—the LASSO and ridge regression, which results in averaging markers that are highly correlated and then using the averaged gene for the model [32]. Random forest (RF) and support vector machines (SVM) are nonparametric models based on supervised learning methods, which have been proved to be effective in detecting interactions between markers [27,33]. Reproducing kernels Hilbert spaces regression (RKHS) is another nonparametric model that is able to capture nonadditive effects [34].

## 3. Factors Affecting Prediction Accuracies of Genomic Selection in Wheat

The prediction accuracy of GS is commonly estimated using cross-validation, in which a set of individuals that are both genotyped and phenotyped is divided into a training set (training population) and validation set (validation population, VP), with marker effects estimated in the training set used to predict GEBVs for the validation set [35]. The accuracy is then measured as the correlation between GEBVs and true breeding values (observed phenotypes) of individuals from the validation set. Prediction accuracy of GS is influenced by various molecular, genetic, and phenotypic factors, as well as the features of the selected statistical model. Genetic factors include the distribution and strength of linkage disequilibrium (LD) between markers and QTL, marker collinearity, population size and structure, differences in allele frequency between TP and VP, etc. [17,30,36,37,38]. Phenotypic factors include factors related to traits themselves, such as heritability and phenotypic variance of the TP [24,39,40]. Other factors that affect the accuracy of the prediction are the number and type of molecular markers, the similarity of the TP and the VP, TP size, and the features of the selected statistical model [28,39,41,42,43,44,45].

Three major factors that affect the GS accuracy are population structure, TP size, and marker density, the effects of which are highly interrelated [46,47]. Population structure can give rise to a false association between a marker and QTL, thus causing structure-generated LD, which can lead to overestimated genomic heritability and biased GS prediction accuracies [37,48]. Meuwissen et al. [49] estimated the minimum number of markers required to reach high prediction accuracy (approximately 0.9) when using unrelated individuals to be equal to 10 times the product of the effective population size and the genome size in Morgans (10 × N_e_ × L), while the minimum size of the population was estimated to be 2 × N_e_ × L. In the case of wheat (the L of which is approximately 30 Morgans) and assumption of N_e_ = 50, that would amount to 15,000 markers and 3000 individuals in an unrelated population. However, those estimations were obtained using simulations, while empirical studies on wheat have reported acceptable accuracies for a much lower number of markers and smaller populations, depending on the population structure [17,21,30]. As in other species, studies on wheat also showed that larger TP reduces bias and decreases the marker effect variance, thus resulting in higher prediction accuracy [31]. The interrelatedness of marker density and population structure seems to play an important role in optimizing GS in wheat. Namely, it has been shown that the higher the relatedness between TP and VP, the smaller the response to increased marker density [48].

## 4. Overview of Genomic Selection Research for Wheat Quality Improvement

The first GS study for wheat quality traits was published in 2011 [21]. The study was conducted in a population composed of multiple wheat families and showed that GS accuracy surpasses MAS accuracy for wheat quality traits by roughly 30% and that GS was about 95% as accurate as PS. Authors also concluded that, regardless of the inferiority when compared to PS, GS has the potential to increase genetic gain per unit of time and costs when applied in a breeding program.

A study by Heffner et al. [30] based on two biparental wheat populations, examined the potential of GS to predict nine wheat quality traits. The authors of the study have found that the mean accuracy obtained by GS was 1.4 times greater than the one obtained by MAS, but that both GS and MAS were inferior to PS. However, those findings were expected due to the polygenic nature and medium to high heritability of all examined traits. Liu et al. [50] reported that, when predicting wheat hybrid performance for seven quality traits, GS extensively outperformed MAS, while giving similar results as PS in the case of higher relatedness of TP and VP. It was only in the case of lower relatedness of TP and VP that GS was preferred over PS, thus emphasizing the importance of additive effects in wheat quality traits. According to Battenfield et al. [51], genetic gain was 1.4 to 2.7 times higher when comparing GS to PS for processing and end-use quality traits since GS requires only marker data and a much larger population can be genotyped than phenotyped for wheat quality traits. Michel et al. [52] investigated the use of GS for predicting dough rheological traits in early generations and proved its substantial benefit over MAS. These findings imply that GS can capture more of the genetic variance of wheat quality traits when compared to MAS since it considers both small effect loci in addition to major QTLs. Nevertheless, all of the above-mentioned studies showed that the accuracy of GS for wheat quality traits is under the influence of many factors, with underlined heritability of the trait, genetic relationship between TP and VP, and size of the TP being the most important driving forces of GS accuracy. A summary of the most relevant GS studies for wheat quality traits, with an overview of factors affecting prediction accuracy covered, is given in Table 1.

### 4.1. Effect of Training Population Size

As early as with the first studies of GS for wheat quality traits, it was demonstrated that TP size (N_TP_) significantly impacts the GS accuracy. The average accuracy for nine wheat quality traits was reported to be roughly 1.6-fold higher for N_TP_ = 96 compared to N_TP_ = 24 when applied to a biparental population [30]. A similar pattern was observed in a study by Heffner et al. [21] in which a population consisting of multiple wheat families was used to predict some quality and agronomic traits. Increasing N_TP_ from 96 to 288 resulted in an overall increase in accuracy by approximately 30%. It is interesting to note that in order to achieve approximately the same GS prediction accuracy, a TP that is three times greater should be used in multifamily populations compared to biparental populations (mean accuracies of 0.58 and 0.52 correspond to N_TP_ = 96 and N_TP_ = 288 in biparental and multifamily population, respectively). The positive influence of an increased number of lines in TP was observed for GPC and protein yield (PY) traits [55], where maximum accuracy was reached at maximum TP size (N_TP_ = 240) and amounted to 0.51 and 0.16 for GPC and PY, respectively. When investigating the influence of using different proportions of the entire population as TP (20–80%), Hu et al. [62] concluded that average prediction accuracy benefited from larger TP size when predicting wheat quality traits such as SDS (sodium dodecyl sulfate) sedimentation volume and thousand-kernel weight (TKW). In agreement with previous studies, Kristensen et al. [59] reported that the highest accuracies were recorded for all examined traits in the case of LOO (leave-one-out) type of cross-validation (the largest possible TP scenario), while the *k*-fold cross-validation proved that the use of smaller TP resulted in slightly lower GS prediction abilities. Similar results for flour yield (FY) and alveograph traits were reported by Kristensen et al. [65].

Overall, the size of the TP depends on the genetic relatedness between TP and VP. The more related the two populations are, the smaller the size of the TP will be needed to obtain satisfying GS prediction accuracies for wheat quality traits [54]. Battenfield et al. [51] also reported enhanced accuracy as a result of increasing TP size and random assignment of full-sibs to TP and VP, therefore, creating a greater genetic relationship. Considering that the phenotyping of wheat quality traits can be both costly and time-consuming, designing a TP that at the same time maximizes genetic diversity and enhances GS accuracy, while being small enough to achieve rapid phenotyping, is key for the successful implementation of GS in a breeding program [53].

### 4.2. Relatedness of Training and Validation Population

As for the other wheat traits [69], it has also been observed in other studies of GS for wheat quality traits that, in order to achieve high GS accuracy, TP and VP have to be closely related. In research by Liu et al. [50] three scenarios with low, intermediate, and high relatedness of TP and VP were created in order to predict seven quality traits of wheat hybrids. As expected, results showed that GS accuracy enhances with an increase of population relatedness, regardless of the prediction model used. However, for the scenario of high relatedness, GS and PS resulted in similar prediction accuracies, suggesting that for highly related populations, PS could be hardly outperformed by GS, whereas in lowly related populations, GS will be a method of choice. Poor prediction accuracies were observed for quality traits in durum wheat when the performance of one population type (doubled-haploid) was predicted based on another population type (breeding panel consisted of varieties and advanced lines) [58]. Kristensen et al. [59] used different types of cross-validations to study the impact of genetic distance of TP and VP on GS prediction accuracy. In LOO cross-validation, the GEBV of each individual is predicted based on the rest of the population, thus representing a scenario where the size of TP and genetic relatedness between TP and VP is as large as possible. Leave-family-out (LFO) cross-validation represents a scenario where different levels of genetic relatedness of TP and VP are present since the GEBV of individuals in each family is predicted based on the remaining families in a given population. Comparing LOO and LFO (lower relatedness), Kristensen et al. [59] concluded that genetic relatedness had a bigger impact on GS accuracy than the size of TP. The predictive abilities decreased the most in the case of GPC (0.5 and 0.2 for LOO and LFO, respectively) when increasing the genetic distance between populations, while the smallest impact of increased genetic distance was recorded in the case of Zeleny sedimentation (0.79 and 0.68 for LOO and LFO, respectively). Similar results were reported for FY and alveograph traits, where the decrease of GS accuracy in a range of 24% to 35% was observed when comparing LOO and LFO cross-validation methods [65], and for Zeleny sedimentation, GPC, TKW, and test weight (TW) [64], suggesting that genetic composition of TP is crucial for achieving accurate genomic predictions.

Prediction accuracies for GPC and PY traits showed a strong bias when predicting within the breeding cycle (lower relatedness) compared to predicting between-cycle (higher relatedness). According to Michel et al. [55], the highest bias for GPC was 86%, whereas PY was overestimated in a range from 17% all the way up to 712%. A study by Juliana et al. [63] has provided evidence that for traits with lower heritability the influence of using lowly related populations will be even more pronounced. Therefore, in order to achieve reliable predictions, the use of a diverse TP is recommended.

### 4.3. Effect of Marker Density

Studies of GS for wheat quality traits investigating the effect of marker density (i.e., number of markers, N_M_) all led to the same conclusion that the accuracy of the prediction enhances with increasing marker density until it reaches a plateau, after which a further increase in marker density has no effect on accuracy [30]. Since required marker density is primarily determined by the extent of LD in the examined population, it is assumed that lower marker density will be sufficient for closely related populations (e.g., biparental population) than for distant populations to achieve satisfying GS prediction accuracy. In a study conducted using two biparental wheat populations [30], average GS prediction reached a plateau at N_M_ = 256, after which a slight drop in accuracy was observed (N_M_ = 384), while in a multifamily approach [21] increasing N_M_ from 192 to 1158 increased GS accuracy by approximately 10%, after which response reached a plateau. Huang et al. [54] reported no significant differences in GS accuracy when using the complete set of markers (N_M_ = 13,198) and a subset of 3919 markers, implying that lower N_M_ is already sufficient for predicting quality traits in wheat elite lines and varieties. Juliana et al. [63] confirmed those findings using subsets of a marker data set that contained less than 70%, 50%, and 10% missing data, which corresponded to a scenario of high coverage (N_M_ = 16,072), moderate coverage (N_M_ = 9285), and low marker coverage (N_M_ = 2253). They concluded that marker density had a minimal impact on GS accuracy, suggesting that when a genomic resolution is reached in a high LD species (i.e., wheat), marker density no longer represents a limiting factor.

The interdependence of marker density and relatedness of TP and VP in the context of GS was illustrated in a study by Liu et al. [50] in which three scenarios representing low, intermediate, and high relatedness were used. In the case of lowly related TP and VP, the plateau was reached after ~3000 markers, whereas in the case of intermediate and highly related TP and VP, the plateau was reached at ~2000 and ~500 markers, respectively.

### 4.4. Effect of Heritability of the Trait

Numerous studies up to date showed that GS accuracy is strongly influenced by heritability, i.e., the fraction of the phenotypic variance of the trait due to genetic variance. Although there is no unambiguous categorization, the majority of studies on wheat categorize heritability values as low (<0.4), moderate (0.4–0.7), and high (>0.7) [21,30]. Generally, traits with high heritability show high GS accuracy and vice versa. The predictive ability of GS for wheat quality traits parallels their heritability which is often showed to be moderate to high. An overview of heritability and GS prediction accuracy ranges reported for some wheat quality traits is given in Table 2.

Studies on wheat quality traits showed that heritability was the main factor that affected the accuracy of GS [61]. Interestingly, not all highly heritable traits showed high GS accuracy. While for most of the highly heritable traits (TW, sucrose solvent retention capacity (Suc-SRC), water solvent retention capacity (H_2_O-SRC), and lactic-acid solvent retention capacity (LA-SRC)), mean GS accuracy across the four models used was 0.6 and higher, for FY and KH, accuracies were 0.45 and 0.38, respectively, despite their heritability values being > 0.9 [54]. Low heritability traits would require larger TP in order to attain the same prediction accuracy as in the case of traits with moderate to high heritability [56]. According to the reported heritabilities (Table 2), it is highly unlikely that the heritability will present a limiting factor in GS for wheat quality traits.

### 4.5. Effect of Model Used

A broad range of models can be used to predict the phenotypic performance of wheat, but the performance of each model is interrelated with the genetic architecture of the examined trait and relatedness of TP and VP. As it is presented in Table 1, the majority of GS studies for wheat quality traits used GBLUP and RRBLUP models, the performance of which was usually compared to one of the Bayesian models.

Little or no difference in prediction accuracy was detected between RRBLUP and Bayesian models in a study by Heffner et al. [21], which suggested that all examined quality traits were controlled by many QTLs of small effect. RRBLUP was comparable by Bayesian models for highly polygenic quality traits in biparental populations while being surpassed in the case of populations with a high genetic variance of examined traits [30]. RRBLUP and BayesCπ showed no significant differences when predicting hybrid performance [50]. BL gave similar or slightly higher prediction accuracies than GBLUP for GPC, TW, TKW, falling number (FN), FY, and alveograph traits, while the biggest difference was recorded in the case of Zeleny sedimentation [59,65]. Those findings may be due to the better performance of Bayesian models in case of lower relatedness of TP and VP, and in case of traits controlled by few major QTLs, since they shrink small effects stronger while shrinking large effects much weaker. Zeleny sedimentation has been proved to be controlled by few QTLs of large effect, hence obtaining higher GS accuracies when Bayesian models were used [59]. Similar results were observed when comparing RRBLUP and BL models for GPC and Zeleny sedimentation [68]. Hu et al. [62] compared two nonparametric (RKHS and RF) and two parametric models (RRBLUP and BL) when predicting SDS sedimentation volume, GPC, and TKW, and concluded that their performance was strongly influenced by prediction scenario (predicting within the same year and across years where years represented different drought conditions). Namely, nonparametric models outperformed parametric in the cross-year prediction which represented a more realistic setting, while in the same-year prediction average performances of RF, RKHS, and RRBLUP were similar, with RF showing significant variations among growing seasons. Only a study by Battenfield et al. [51] showed that when GS accuracy was obtained by cross-validation, Gaussian kernel (GAUSS) was the best model for predicting all quality traits within a population consisting of multiple families, thus outperforming EN, partial least square regression (PLSR), and RRBLUP.

Bayesian models usually require longer computation time compared to GBLUP or RRBLUP [12,58,62] but show no clear superiority over the other models across wheat quality traits [61,68], i.e., the accuracy of GS for wheat quality traits was generally not under the large influence of prediction model applied. Therefore, RRBLUP showed to be a model of choice in the majority of GS studies for wheat quality traits [54,58] due to its robustness and shorter computational time [55].

## 5. Multitrait Genomic Selection

Wheat quality traits can often be hard to improve, since they usually require a large amount of flour and/or labor to be invested, thus limiting the size of the TP that can be phenotyped which leads to insufficient GS accuracy. Incorporating additional phenotypic information in the multitrait approach for GS could help to overcome the problem of potentially low GS accuracy obtained for wheat quality traits. Multitrait GS data obtained utilizing rapid quality tests are used for predicting parameters of more laborious wheat quality tests. Rapid tests such as near-infrared (NIR) and nuclear magnetic resonance (NMR) methods are less labor-intensive and require a small amount of flour. It has been proved that incorporating NIR and NMR data into the multitrait approach increases the accuracy of GS for some wheat quality traits (accuracy ranged between 0 and 0.47, and between 0 and 0.69 in a single-trait approach and multitrait approach, respectively) [56]. Incorporating easily obtained gluten peak indices into multitrait GS analysis improved average prediction accuracy by roughly 20% in comparison to single-trait GS for dough rheology traits [57]. Including metabolomics data in GS resulted in increased accuracies for some wheat quality traits (GPC, GC, FN, FY, Zeleny sedimentation, KH) compared to GS based on DArT markers only [71]. According to Haile et al. [58], the multitrait approach resulted in higher prediction accuracy only in the case of yield, whereas for quality traits, all single-trait models applied gave better prediction accuracy compared to the multitrait approach. Lado et al. [60] showed that no multitrait model used performed better than a single-trait model, but that using highly correlated traits in multitrait GS for wheat quality allows reduction of TP up to 30% without significantly affecting the predictive ability of the model. Further research studies showed that using different GS indices in simultaneous selection for yield and wheat quality traits still does not outperform single-trait prediction for GPC, PY, and the dough rheological traits, but suggested that simultaneous improvement of yield and wheat quality should target protein quality, rather than GPC [66]. A significant gain of multitrait approach is expected only for low heritable traits that are incorporated with high heritable traits, between which high genetic correlation exists [64]. Data for traits incorporating together in a multitrait analysis must be already available or easy to obtain on a large number of samples in a short period of time [67].

## 6. Conclusions

Due to the complex nature of inheritance for the majority of wheat quality traits, GS seems to be the method of choice because it simultaneously accounts for small and medium effect loci as well as for major QTLs. Numerous studies in the last decade proved that GS has sufficient accuracy for implementation in the breeding programs targeting wheat quality. Genomic selection can be helpful in predicting the performance of lines in early generations and preselecting high-performing lines, boosting trait stability, and efficiently selecting superior genotypes for wheat quality traits. There is some evidence that GS could also be used to address one of the biggest problems in wheat breeding—how to simultaneously select for grain yield and quality traits since the existence of a strong negative correlation between those traits is well known and documented. Nevertheless, before implementing GS in the breeding for wheat quality traits, some limitations considering trait heritability, genetic relationship between TP and VP, and size of the TP must be taken into account.

## Figures and Tables

**Table 1:** Overview of references for genomic selection studies covering wheat quality traits.

Reference	Quality Traits Examined ^1^	Population Type and Size ^2^	Platform and Number of Markers ^2^	GS Prediction Model ^3^	Factors Affecting Prediction Accuracy Examined ^2^				Comparison to Other Types of Selection ^2^	Single-Trait (ST) or Multitrait (MT) Analysis
					Selected Model	TP Size	TP/VP Relatedness	Marker Density		
[30]	TW, PHS, FY, KH, LA-SRC, NaCO-SRC, Suc-SRC, H_2_O-SRC	2 biparental populations (209/174 DHs)	399 multiple platforms/574 DArTs	RRBLUP, BayesCπ	Yes	Yes	No	Yes	Yes (MAS and PS)	ST
[21]	TW, PHS, FY, FP, LA-SRC, NaCO-SRC, Suc-SRC, H_2_O-SRC, KH	374 lines	1158 DArTs	RRBLUP, BayesA, BayesB, BayesCπ	Yes	Yes	No	Yes	Yes (MAS and PS)	ST
[51]	TKW, TW, GPC, FY, FP, LV, KH, SDS sedimentation, mixograph and alveograph traits	5520 lines	3075 SNPs	RRBLUP, GAUSS, PLSR, EN, RF	Yes	Yes	No	No	Yes	ST
[53]	TKW, TW, GPC, KH, SDS sedimentation	8416/2403 landrace accessions	~23,000/~33,000 DArT SNPs	GBLUP	No	Yes	Yes	No	No	ST
[54]	TW, FY, FP, LA-SRC, NaCO-SRC, Suc-SRC, H_2_O-SRC, KH	273 elite lines and cultivars	3919/13,198 SNPs	RRBLUP, BRR, RKHS, EN	Yes	Yes	Yes	Yes	No	ST
[50]	TKW, TW, GPC, GC, SC, KH, Zeleny sedimentation	135 inbred lines,1604 hybrids	17,372 SNPs	RRBLUP, W-BLUP, BayesCπ	Yes	Yes	Yes	Yes	Yes (PS)	ST
[55]	GPC, PY	659 lines	9500 DArT SNPs	RRBLUP	No	Yes	Yes	No	No	ST
[56]	TKW, TW, GPC, FY, FP, SC, amylose content, FN, LV, LT, MIXT, KH, starch damage, viscosity, farinograph and extensograph traits	2076 varieties and synthetic derivative lines	51,208 SNPs	Multivariate model	No	No	No	No	No	ST + MT
[57]	GPC, farinograph, extensograph, and alveograph traits	128 DHs	6600 DArT SNPs	RRBLUP	No	No	No	No	No	MT
[58]	GPC, gluten index, alveograph traits	170 varieties and advanced lines, 154 DHs	9752/5153 SNPs	RRBLUP, GBLUP, BayesA, BayesB, BL, RKHS, MT-BayesA, MT-Matrix, MT-SI	Yes	No	Yes	Yes	No	ST + MT
[59]	TKW, TW, GPC, FN, Zeleny sedimentation	635 lines (159 full-sib families)	10,802 SNPs	GBLUP, BL	Yes	Yes	Yes	Yes	No	ST
[60]	TW, GPC, WGC, SV, alveograph and mixograph traits	495 lines	6655 SNPs	BRR, Bayes multivariate Gaussian model	No	Yes	No	No	No	ST + MT
[52]	GPC, farinograph and extensograph traits	840 lines	4598 DArT SNPs	RRBLUP, W-BLUP	Yes	No	No	No	Yes (MAS)	ST + MT
[61]	TKW, GPC, mixograph, farinograph, and extensograph traits	57 cultivars and lines	7588 SNPs	RRBLUP, BayesA BayesB, BL, BRR	Yes	No	No	No	No	ST + MT
[62]	TKW, GPC, SDS sedimentation	282 DHs	7426 SNPs	RRBLUP, BL, RF, RKHS	Yes	Yes	No	No	Yes (PS)	ST
[63]	TKW, TW, GPC, FY, FP, FS, LV, MIXT, KH, grain color, alveograph traits	3485 lines	78,606 SNPs	GBLUP, BayesB	Yes	No	Yes	Yes	No	ST
[64]	TKW, GPC, FN, Zeleny sedimentation	1152 lines	11,058 SNPs	GBLUP, Bayesian SNP-BLUP	Yes	Yes	Yes	No	Yes (MAS)	ST + MT
[65]	FY, alveograph traits	635 lines (159 full-sib families)	10,802 SNPs	GBLUP, BL	Yes	Yes	Yes	No	No	ST
[66]	GPC, PY, extensograph and farinograph traits	480 lines	7300 DArT SNPs	GBLUP, W-BLUP	Yes	No	No	No	No	ST + MT
[67]	TKW, TW, GPC, FP, LV, KH, SDS sedimentation, mixograph and alveograph traits	~1400 lines	78,606 SNPs before filtering *	BMTME, MTR	Yes	No	No	No	No	MT
[68]	GPC, Zeleny sedimentation	1325 lines	9290 SNPs	RRBLUP, BL	Yes	No	No	No	No	ST

* Final number of markers used for analysis is not mentioned. ^1^ TKW—thousand-kernel weight, TW—test weight, GPC—grain protein content, FY—flour yield, FP—flour protein, FS—flour sedimentation, WGC—wet gluten content, PY—protein yield, GC—gluten content, KH—kernel hardness, SC—starch content, FN—falling number, LV—loaf volume, LT—loaf texture, MIXT—mixing time, SV—sedimentation volume, PHS—preharvest sprouting, LA-SRC—lactic acid solvent retention capacity, NaCO-SRC—sodium carbonate solvent retention capacity, H_2_O-SRC—water solvent retention capacity, Suc-SRC—sucrose solvent retention capacity, SDS—sodium dodecyl sulfate. ^2^ DH—double haploid, SNP—single nucleotide polymorphism, TP—training population, VP—validation population, PS—phenotypic selection, MAS—marker-assisted selection, ST—single-trait, MT—multitrait. ^3^ GS—genomic selection, RRBLUP—ridge regression best linear unbiased prediction, GBLUP—genomic best linear unbiased prediction, BL—Bayesian least absolute shrinkage and selector operator (LASSO), BRR—Bayesian ridge regression, GAUSS—Gaussian kernel, PLSR—partial least squares regression, RKHS—reproducing kernel Hilbert space, EN—elastic net, W-BLUP—weighted best linear unbiased prediction, BMTME—Bayesian multitrait multienvironment, MTR—multitrait ridge regression, MT-SI—multitrait selection index, RF—random forest.

**Table 2:** Overview of heritability and GS prediction accuracy reported in studies covering wheat quality traits.

Reference	Quality Traits Examined ^1^	Heritability Type	Heritability Strength	Heritability Range	GS Prediction Accuracy Range ^3^
[21]	PHS, GPC, TW, Suc-SRC, LA-SRC, KH, FY	broad-sense	high	0.71–0.93	0.45–0.76
[30]	TW, PHS, FY, KH, LA-SRC, NaCO-SRC, Suc-SRC, H_2_O-SRC	broad-sense	moderate—high	0.67–0.95	0.27–0.74
[51]	TKW, TW, GPC, FY, FP, SDS sedimentation, KH, LV, mixograph and alveograph traits	narrow-sense	moderate	0.41–0.68	0.42–0.71
[54]	TW, FY, FP, KH, LA-SRC, NaCO-SRC, Suc-SRC, H_2_O-SRC	alternative calculation for unbalanced data ^2^	high	0.75–0.95	0.31–0.67
[50]	TKW, TW, GPC, GC, SC, KH, Zeleny sedimentation	broad-sense	moderate—high	0.63–0.96	0.35–0.96 ^4^
[57]	GPC, farinograph, extensograph, and alveograph traits	alternative calculation for unbalanced data ^2^	moderate—high	0.69–0.83	0.16–0.61 ^4^
[59]	TKW, TW, GPC, FN, Zeleny sedimentation	narrow-sense	moderate—high	0.56–0.81	0.2–0.79
[60]	TW, GPC, WGC, SV, alveograph and mixograph traits	broad-sense	moderate	0.36–0.64	0.24–0.43 ^4^
[52]	GPC, farinograph and extensograph traits	narrow-sense	moderate	0.4–0.66	0.3–0.53
[61]	TKW, GPC, mixograph, farinograph, and extensograph traits	broad-sense	high	0.78–0.93	0.25–0.42
[65]	FY, alveograph traits	narrow-sense	moderate—high	0.38–0.72	0.3–0.79
[68]	GPC, SC, Zeleny sedimentation	narrow-sense	low—moderate	0.35–0.62	0.1–0.3

^1^ TKW—thousand-kernel weight, TW—test weight, GPC—grain protein content, FY—flour yield, FP—flour protein, WGC—wet gluten content, GC—gluten content, KH—kernel hardness, SC—starch content, FN—falling number, LV—loaf volume, SV—sedimentation volume, PHS—preharvest sprouting, LA-SRC—lactic acid solvent retention capacity, NaCO-SRC—sodium carbonate solvent retention capacity, H_2_O-SRC—water solvent retention capacity, Suc-SRC—sucrose solvent retention capacity, SDS—sodium dodecyl sulfate. ^2^ According to Piepho and Möhring [70]. ^3^ Accuracy across all used models or scenarios. ^4^ Accuracy of single-trait genomic selection model.

## Data Availability

Not applicable.
